# The Effects of International Trade on Water Use

**DOI:** 10.1371/journal.pone.0132133

**Published:** 2015-07-13

**Authors:** Kazuki Kagohashi, Tetsuya Tsurumi, Shunsuke Managi

**Affiliations:** 1 Graduate School of Economics, Kyoto University, Kyoto, Japan; 2 Department of Policy Studies, Nanzan University, Nagoya, Japan; 3 Department of Urban and Environmental Engineering, Kyushu University, Fukuoka, Japan; 4 QUT Business School, Queensland University of Technology, Brisbane, Australia; Universita' del Piemonte Orientale, ITALY

## Abstract

The growing scarcity of water resources worldwide is conditioned not only by precipitation changes but also by changes to water use patterns; the latter is driven by social contexts such as capital intensity, trade openness, and income. This study explores the determinants of water use by focusing on the effect of trade openness on the degree to which water is withdrawn and consumed. Previous studies have conducted analyses on the determinants of water use but have ignored the endogeneity of trade openness. To deal with this endogeneity problem, we adopt instrumental variable estimation and clarify the determinants of water use. The determinants of water use are divided into *scale*, *technique*, and *composition effects*. Calculating each trade-induced effect, we examine how trade openness affects the degree of water use. Our results show that while trade has a positive effect on water withdrawal/consumption through trade-induced scale effects and direct composition effects, the trade-induced technique and the indirect composition effect, both of which exhibit a negative sign, counteract the scale effect and the direct composition effect, resulting in reduced water withdrawal/consumption. The overall effect induced by trade is calculated as being in the range of –1.00 to –1.52; this means that the overall effect of a 1% increase in the intensity of trade openness reduces the degree of water withdrawal/consumption by roughly 1.0–1.5%, on average. This result indicates that international bilateral trade would promote efficient water use through the diffusion of water-saving technologies and the reformation of industry composition.

## Introduction

Water is essential to human survival and well-being. It is argued that each individual requires at least 5 l/day for survival and 50 l/day to live comfortably [1]. Water can also be a scarce resource, as it is used for such widespread activities as irrigation in agricultural production, cooling and cleaning in industrial production, and power generation. Water resources have become increasingly scarce worldwide: global warming has disrupted regional water cycles, and the total area of regions affected by drought (e.g., the Sahel, the Mediterranean, southern Africa, and parts of southern Asia) has likely increased since the 1970s [2]. Moreover, socioeconomic factors such as global population growth, high standards of living, increasing urbanization, and the expansion of irrigated agriculture has caused a nearly seven-fold increase in freshwater withdrawals between 1900 and 2000 [3], [4]. Among these socioeconomic factors, irrigated agriculture accounts for approximately 80% of global water withdrawals [5]. The amount of water required for irrigation depends on, for example, physiographic conditions, irrigation techniques, and the serviceable conditions of irrigation systems [6]. The international trade of commodities is another critical factor that affects water scarcity, since export goods (grains in particular) consume a certain amount of what is called “virtual water” [7] in the production process. Approximately 1,000 m^3^ of water is required to produce 1 ton of grain, for example [8]. Thus, water scarcity is influenced by not only precipitation changes but also by the pattern of water use, which is driven by social contexts such as capital intensity (the capital–labor ratio), trade openness, and income. This study explores the determinants of water use while focusing on the effect trade openness has on them.

Researchers have analyzed how trade and income affect water withdrawal [9], [10], [11], [12]. As to the determinants of water use, recent studies [10], [12] have investigated the relationship between income and the degree of water consumed or withdrawn. Cole [10] analyzed the relationship between per-capita water consumption (dependent variable) and income, using panel data [6] for 40 countries; he confirms a statistically significant inverted-U relationship between water consumption and income. Hoehn and Adanu [12] also tested an inverted-U relationship between water utilization and income, using the International Hydrological Programme (IHP) database [13] containing data from 32 countries for 1970, 1980, and 1990. The dependent variables in that study were water withdrawal and consumption, and the independent variables were 1) economic scale, 2) capital intensity, 3) trade openness, 4) income (and its squared term), 5) temperature (and its squared term), 6) precipitation, and 7) climate dummies. They prepared two econometric models. One was the environmental Kuznets curve (EKC) model, from which the variables of economic scale, capital intensity (capital–labor ratio), and trade openness were omitted; they found no support for the EKC. The other model was the two-sector trade model, which extends the EKC model by incorporating the omitted variables above. Using generalized least squares estimation, they found that capital intensity, trade openness, and income might have negative effects on water use, while the economic scale tends to increase it; this conclusion provides no support for the EKC. As they treated income and trade openness as exogenous, however, the ordinary least squares estimator may have led to biased and inconsistent results; hence, the overall impact of trade and income on water use remains inconclusive. Using an analytical framework to deal with the endogeneity problem [14], [15], we analyze the causal effects of trade openness on water withdrawal or consumption.

As this study focuses on the relationship between trade openness and water use, studies that analyze the effect of the “virtual water trade” may be relevant. The concept of “virtual water,” first proposed in the 1990s, has been developed to explain how physical water scarcity in the Middle East is relaxed by importing water-intensive commodities such as cereals, grains, and meats [16]. Using 1995 data on the global cereal trade, De Graiture et al. reveal that 112 km^3^ of water resources—which is equivalent to 11% of the world’s irrigation water—were saved by exporting cereals from trade partners with a comparative advantage in agriculture (e.g., the United States, the European Union, Canada, Australia, and India) to cereal-importing countries (e.g., Japan, China, Pakistan, South Korea, and Egypt) [17]. This result is also supported by economic theory [18]. Using the Heckscher–Ohlin trade model, the level of water consumption invariably decreases once water-abundant and water-scarce countries start to trade [18]. Specifically, Antweiler [19] pioneered the concept of “virtual water trade,” which is essentially based on the notion of pollution embodied in trade; this concept embodies the essence of the Heckscher–Ohlin model. This concept has been widely accepted and analyzed in the literature [20], [21], [22], [23]; such studies have helped clarify the volume of water we use in trading commodities and understand the physical accounting of water resources. However, little attention has been paid to the net effect of trade liberalization on water use. This paucity is a limitation of the existing virtual water trade literature, and our study aims to address it by examining the impact of promoting trade openness on water withdrawal or consumption.

## Materials and Methods

### Data

We obtained water withdrawal and consumption data [13] from 79 countries for the years 1960, 1970, 1980, 1990, 1995, and 2000. Here, “water withdrawal” refers to the amount of water withdrawn from rivers, lakes, groundwater, and the like, irrespective of whether or not the water will be returned to the original reservoir or water system after its use. On the other hand, “water consumption” refers to the amount of water that people cannot easily reuse, due to evaporation, transpiration by plants, incorporation into crops, consumption by people or livestock, and the like. Unfortunately, the initial dataset was limited to 50 countries; therefore, we used water resources data from another database [24] that covers the countries that appear in the database of Shiklomanov [13]. Hydrological variables such as annual mean precipitation and annual mean temperature were calculated using *The Global Historical Climatology Network-Monthly (GHCN-M) Version 2* database [25]. Using these hydrological variables and data on monthly precipitation and temperature, we introduced dummy variables that represent each country’s hydrological conditions; we also classified the 79 countries into five groups based on the Koppen’s climate classifications (i.e., A, B, C, D, and E), as per Peel et al. [26]. If the temperature in a country’s coldest month is 18°C or higher, it is classified as A (“Tropical”); if the temperature in a country’s hottest month is lower than 10°C, it is classified as E (“Polar”). In our analysis, we found 29 countries to be of classification A, and no countries were in classification E. We also found 16 countries to be of classification B (“Arid”); these were the countries whose mean annual precipitation is less than 10 times a threshold value that depends upon the relationship between the distribution of annual precipitation and the mean annual temperature. (For details on determining that threshold value, see Peel et al. [26].) Classifications C and D denote “Temperate” and “Cold,” respectively; the former corresponds to those countries where the temperature in the hottest month is higher than 10°C, while that of the coldest month is between 0°C and 18°C. The latter, meanwhile, corresponds to countries where the temperature in the hottest month is higher than 10°C, but that of the coldest month is not above 0°C. We found 19 countries within our dataset to be of classification C, while 15 countries were of classification D.

Economic data were drawn from various sources. Real gross domestic product (GDP) data were obtained from *Penn World Table 7*.*0*, bilateral trade flows from the *CIA World Factbook*, capital–labor ratios from *Extended Penn World Tables 4*.*0*, and country-specific data (used in the trade equation in section 2.3) from the *CIA World Factbook*. The dataset comprising data from the four aforementioned sources included 196 countries from 1950 to 2006. In the calculation of total factor productivity (TFP) in section 2.4, we use *Extended Penn World Tables* for data regarding capital stock and the number of employed workers; these data cover 117 countries from 1963 to 2009. Agricultural output data, which are required for the calculation of its proportion to GDP and used in the estimation of the water use equation in section 2.5, were taken from *World Development Indicators*.

**Fig 1:**
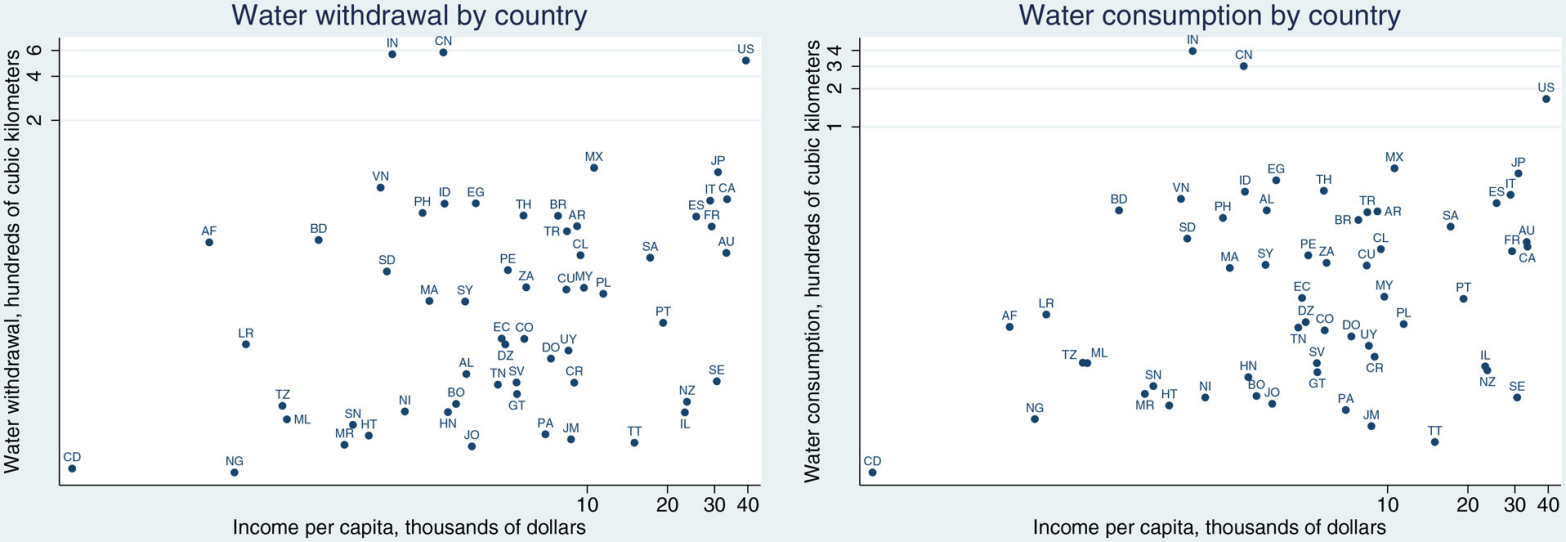
Water withdrawal and consumption by country. The scatter plot as appeared on the left side of the diagram illustrates the relationship between each country’s water withdrawal and per capita income while right side of the diagram demonstrates the relationship between each country’s water consumption and per capita income. On each plot, country names are labeled with the ISO-3166 codes.

The variables we use in this study and their sources are summarized in Table 1. Fig 1 demonstrates the relationship between each country’s water withdrawal/consumption and per capita income.

**Table 1:** List of variables.

Variable name	Variable Label	Source of the data
*Trade*_*ijt*_	Bilateral trade flows from country i to country j, measured in US$	*Direction of Trade Statistics*
*Y*_*it*_	Real gross domestic product, measured in US $billion	*Penn World Table 7*.*0*
*Dis*_*ij*_	Distance between countries between country i and j	*CIA World Factbook*
*Lan*_*ij*_	Language dummy if country i and j have a common language	*CIA World Factbook*
*Bor*_*ij*_	Border dummy if country i and j have a common border	*CIA World Factbook*
*Landlocked*_*ij*_	Landlocked dummy if both country i and j are landlocked	*CIA World Factbook*
*Z*_*it*_	Total factor productivity (TFP)	Calculated by authors
*RZ*_*it*_	TFP relative to the world average	Calculated by authors
*K*_*it*_	Estimated net fixed standardized capital stock in 2005 purchasing power parity	*Extended Penn World Tables 4*.*0*
*L*_*it*_	Number of employed workers	*Extended Penn World Tables 4*.*0*
*w*_*it*_	Annual water withdrawal in country i (billion cubic meters)	*World Development Indicators*
*c*_*it*_	Annual water consumption in country i (billion cubic meters)	*World Development Indicators*
*T*_*it*_	Trade openness	Calculated by authors
*(K/L)*_*it*_	Capital-labor ratio ($1,000 per worker)	*Extended Penn World Tables 4*.*0*
*(RK/L)*_*it*_	Capital-labor ratio relative to the world average	Calculated by authors
*(Agri)*_*it*_	Percentage of agricultural output in GDP	Calculated by authors (agricultural output data is obtained from *World Development Indicators*)
*(RWs)*_*it*_	Relative water abundance index defined as the ratio of water abundance index (see (*Ws)*_*it*_) to the world average	Calculated by authors
*(Ws)*_*it*_	Water abundance index defined as the ratio of the amount of water withdrawal/consumption to the water resources available in country i	Calculated by authors
*Prcp*_*it*_	Average annual precipitation	*The Global Historical Climatology Network-Monthly (GHCN-M) ver*.*2*
*Temp*_*it*_	Average annual temperature	*The Global Historical Climatology Network-Monthly (GHCN-M) ver*.*2*
*Tropical*_*i*_	Tropical dummy if country i is located in a tropical climate zone	Calculated by authors
*Dry*_*i*_	Dry dummy if country i is located in a dry climate zone	Calculated by authors
*Cold*_*i*_	Dry dummy if country i is located in a cold climate zone	Calculated by authors
*Area*_*i*_	Land area (millions of km^2^)	*CIA World Factbook*

### Statistical methods

Four equations are considered in our study—namely, a trade equation, a TFP equation, a growth equation, and a water use equation. For the first, we adopt a Poisson pseudo-maximum-likelihood (PPML) estimation method, since the PPML estimator behaves well under conditions that feature zero trades [27]. In line with previous empirical studies [14], [28], [29], [30], we construct an instrumental variable for trade openness based on what is called the “gravity equation”—an empirical model considered one of the most successful in explaining the determinants of bilateral trade flow between countries *i* and *j*. We compute the instrumental variable for trade openness by taking the exponents of the fitted values of bilateral trade and aggregating them across bilateral trading partners (countries *i* and *j*). To maintain consistency of estimation, we estimate all equations other than the trade equation by using two-step generalized method of moments. From the results of the estimation of the trade equation, we construct an instrumental variable of trade openness and use it in the TFP equation. We then construct the instrumental variable of TFP and estimate the growth equation by using it. This method is also applied to the water use equation—which is to say, we estimate the equation with the previously estimated instrumental variables of trade openness, TFP, and GDP.

### Trade equation

It is widely acknowledged that the ratio of trade flow from country *i* to country *j* to GDP (trade openness) is determined by indicators of each country’s size of land area and of the distance between them—including physical distance, linguistic commonality (or its lack), the existence of a common border (or lack thereof), and any landlock. We have chosen these variables, in line with Frankel and Romer [30] and Silva and Tenreyro [31] in the gravity equation.

$$Trade_{ijt}=c_{1}+\alpha_{1}\text{ln}Y_{it}+\alpha_{2}\text{ln}Y_{jt}+\alpha_{3}\text{ln}Dis_{ij}+\alpha_{4}Lan_{ij}+\alpha_{5}Bor_{ij}+\alpha_{6}Landlocked_{ij}+\eta_{1i}+\upsilon_{1it},$$ (1)

where *Trade*_*ijt*_ is bilateral trade flows from country *i* to country *j*, defined as the sum of the aggregate exports and imports between these countries; *Y*_*it*_, *Y*_*jt*_ are the real GDP of countries *i* and *j*, respectively; *Dis*_*ij*_ is the distance between countries *i* and *j*; and *Lan*_*ij*_ and *Bor*_*ij*_ are, respectively, the dummy variables denoting whether country *i* and *j* share a common language or border. *Landlocked*_*ij*_ is a dummy that takes the value of 2 if both countries are landlocked, 1 if one country is landlocked, and 0 otherwise; *η*_1_ is an individual country effect; and *υ*_1_ is a random disturbance. Note that we estimate the trade equation using the PPML regression method—the estimator of which, as mentioned, behaves well even with zero trades. Using the estimation result, we construct the instrumental variable of trade openness (${\hat{T}}_{it}$) as $\sum_{j}\left({\tilde{T}}_{ij}/Y_{i}\right)$, where ${\tilde{T}}_{ij}$ is a predicted value of *Trade*_*ijt*_.

### Total factor productivity equation and growth equation

The use of water-related technologies such as drip irrigation and water recycling would naturally affect a country’s use of water. We capture this technique effect through the use of TFP. The TFP equation is specified as follows:

$$Z_{it}=c_{2}+\beta_{1}{\hat{T}}_{it}+\eta_{2i}+\upsilon_{2it},$$ (2)

where *Z*_*it*_ is the level of TFP of country *i* in year *t*, ${\hat{T}}_{it}$ is trade openness, *η*_2_ is an individual country effect, and *υ*_2_ is a random disturbance.

Following [32], [33] and [34], we calculate the level of TFP in each country by way of the following equation:

$$Z_{it}=\frac{Y_{it}}{K_{it}^{\bar{\alpha }}H_{it}^{1-\bar{\alpha }}},$$ (3)

where *K*_*it*_ and *H*_*it*_ are, respectively, the net fixed standardized capital stock in 2005 purchasing power parity and the number of employed workers in country *i* in year *t*. Following the specification in [32] and [33], the level of human capital is specified as *H*_*it*_ = *L*_*it*_ *e* ^*ϕ*(*s*)^, where *L*_*it*_ is the number of employed workers and *e* ^*ϕ*(*s*)^ is the efficiency of a unit of labor with years of school enrollment. *s* is the average years of schooling and *ϕ*(*s*) is a linear function with a slope of 0.134 for *s* ≤ 4, 0.101 for 4 < *s* < 8, and 0.068 for *s* ≥ 8, based on the result of an empirical analysis of the rate of returns to investment in education [35]. Specifically, following the equation in [33], we have *ϕ*(*s*) = 0.134 × *s* if *s* ≤ 4; *ϕ*(*s*) = 0.134 × 4 + 0.101 × (*s*—4) if 4 < *s* < 8; and *ϕ*(*s*) = 0.134 × 4 + 0.101 × 4 + 0.068 × (*s*—8) if *s* ≥ 8. $\bar{\alpha }$ is a constant and presumed to be $\bar{\alpha }=1/3$, following the argument in [36].

Using the predicted TFP value (denoted as ${\hat{Z}}_{it}$) in Eq (2), we also estimate a simple growth regression model:

$$\text{ln}Y_{it}=\text{ln}c_{3}+\delta_{1}\text{ln}K_{it}+\delta_{2}\text{ln}L_{it}+\delta_{3}\text{ln}{\hat{Z}}_{it}+\varepsilon_{3it}.$$ (4)

The coefficient of ln ${\hat{Z}}_{it}$ is needed to calculate the elasticity of trade openness; this takes place in section 2.6.

### Water use equation

In line with previous studies on the empirical specification of trade openness interactions with a comparative advantage [37], [38]—and in particular, in line with Managi et al. [14]—we specify the water use equation as follows:

$$\begin{array}{c} w_{it}=c_{4}+\gamma_{1}{\hat{Y}}_{it}+\gamma_{2}{\hat{Z}}_{it}+\gamma_{3}{\hat{Z}}_{it}^{2}+\gamma_{4}{\left(K/L\right)}_{it}+\gamma_{5}{\left(K/L\right)}_{it}^{2}+\gamma_{6}{\left(K/L\right)}_{it}{\hat{Z}}_{it}\\ +\gamma_{7}{\hat{T}}_{it}+\gamma_{8}{\left(RK/L\right)}_{it}{\left(RWs\right)}_{it}{\hat{T}}_{it}+\gamma_{9}{\left(RK/L\right)}_{it}^{2}RWs_{it}{\hat{T}}_{it}+\gamma_{10}RZ_{it}{\hat{T}}_{it}\\ +\gamma_{11}RZ_{it}^{2}{\hat{T}}_{it}+\gamma_{12}{\left(RK/L\right)}_{it}RZ_{it}RWs_{it}{\hat{T}}_{it}+\gamma_{13}Prcp_{it}+\gamma_{14}Temp_{it}\\ +\gamma_{15}Temp_{it}^{2}+\gamma_{16}Tropical_{i}+\gamma_{17}Dry_{i}+\gamma_{18}Cold_{i}+\gamma_{19}Area_{i}+\eta_{4i}+\upsilon_{4it} \end{array}$$ (5)

where *w*_*it*_ denotes the degree of water withdrawal or water consumption in country *i* in year *t*, and ${\hat{Y}}_{it}$ is the predicted GDP value of that country in year *t*. ${\hat{Z}}_{it}$ denotes country *i*’s predicted value of TFP level, and *RZ*_*it*_, the level of predicted TFP relative to the world average. Similarly, *(K/L)*_*it*_ denotes country *i*’s capital–labor ratio and *(RK/L)*_*it*_, the relative capital–labor ratio. ${\hat{T}}_{it}$ denotes the predicted value of trade openness, which is defined as the ratio of aggregate exports and imports to GDP. (*RWs)*_*it*_ is a water abundance index (see below).

Our specification is based on that in [14], but has some explicit differences. First, we do not take income as a proxy for the technological level of a country and focus on it more directly by using the TFP variable. Second, we add an index *(Ws)*_*it*_ that denotes country *i*’s degree of water abundance (or, equivalently, water scarcity) in year *t* to capture any comparative advantage in terms of water resources. *(Ws)*_*it*_ is defined as the ratio of the amount of water withdrawal or consumption to the water resources available in country *i* in year *t*. We then define the relative water abundance as:

$${\left(RWs\right)}_{it}=\frac{{\left(Ws\right)}_{it}}{\sum_{i=1}^{n}{\left(Ws\right)}_{it}/n}.$$ (6)

As we presume that the degree of water abundance relates to the comparative advantage of a country, we put the relative water abundance index in the interaction term of the relative capital–labor ratio. Finally, we dropped a lagged term from the water use equation, as in this study we concentrate on the short-term effect of trade openness.

*Prcp*_*it*_ and *Temp*_*it*_ are the average annual precipitation and average annual temperature of country *i* in year *t*, respectively. Following Hoehn and Adanu [12], we add a squared term of average annual temperature; additionally, note that the squared term of average annual precipitation is not included in Eq (1). *Area*_*i*_ denotes land area. We add this term to capture the degree of water abundance, because larger countries tend to have more diverse and plentiful water resources due to their larger geographic diversity (e.g., small islands tend to have only a small fraction of groundwater, while large countries have not only a fair amount of groundwater, but also other sources of water, such as rivers and lakes). *Tropical*_*i*_, *Dry*_*i*_, and *Cold*_*i*_ are the dummy variables of country *i*: *Tropical*_*i*_ is a tropical dummy that takes the value of 1 if the country is located in a tropical zone, and 0 otherwise; similarly, *Dry*_*i*_ and *Cold*_*i*_ are the dry and cold dummy variables, respectively, where 1 indicates that the country is located in a dry or cold zone, and 0 indicates otherwise. The GDP term and climate variables are added to take into account the scale effect and climate conditions. *η*_4_ is an individual country effect and *ν*_4_ is a random disturbance.

We specify another form of the water use equation where the capital–labor ratio variable (*K/L*) is replaced by the proportion of agricultural output to GDP (denoted as *Agri*), as follows:

$$\begin{array}{c} w_{it}=c_{4}+\gamma_{1}{\hat{Y}}_{it}+\gamma_{2}{\hat{Z}}_{it}+\gamma_{3}{\hat{Z}}_{it}^{2}+\gamma_{4}{\left(Agri\right)}_{it}+\gamma_{5}{\left(Agri\right)}_{it}^{2}+\gamma_{6}{\left(Agri\right)}_{it}{\hat{Z}}_{it}\\ +\gamma_{7}{\hat{T}}_{it}+\gamma_{8}{\left(RK/L\right)}_{it}{\left(RWs\right)}_{it}{\hat{T}}_{it}+\gamma_{9}{\left(RK/L\right)}_{it}^{2}RWs_{it}{\hat{T}}_{it}+\gamma_{10}RZ_{it}{\hat{T}}_{it}\\ +\gamma_{11}RZ_{it}^{2}{\hat{T}}_{it}+\gamma_{12}{\left(RK/L\right)}_{it}RZ_{it}RWs_{it}{\hat{T}}_{it}+\gamma_{13}Prcp_{it}+\gamma_{14}Temp_{it}\\ +\gamma_{15}Temp_{it}^{2}+\gamma_{16}Tropical_{i}+\gamma_{17}Dry_{i}+\gamma_{18}Cold_{i}+\gamma_{19}Area_{i}+\eta_{4i}+\upsilon_{4it} \end{array}$$ (7)

In Eq (7), we adopt the *Agri* variable as a proxy for the capital–labor ratio (*K/L*). This is because *K/L* cannot distinguish between primary and tertiary industries, though it is useful for bringing into focus the difference between primary and secondary industries. If we assume that water resources are used extensively in primary industries, it would be reasonable to focus on the proportion of agricultural output to GDP, in addition to the capital–labor ratio. Here, we adopt the capital–labor ratio relative to the world average (i.e., *RK/L*) as a proxy of the comparative advantage of a country. It may be possible to think of another variable—for example, the share of agricultural output relative to the global average. However, it is not clear whether this variable would capture the comparative advantage. Hence, we use *RK/L*, which appears in both Eqs (5) and (7). This treatment is theoretically consistent with the Heckscher–Ohlin model, which shows that the trade equilibrium can be determined by the relative factor endowment by country.

### Scale, technique, and composition effects

The determinants of water use can be decomposed into scale, technique, and composition effects [39]. The scale effect refers to the effect of an increase in GDP on the degree of water use. The technique effect denotes the negative impact of TFP on the degree of water use. It is presumed here that efficient water-use technologies (e.g., drip irrigation systems or irrigation pipes with low rates of leakage) are likely to be introduced as the TFP level increases. The composition effect denotes how the industrial structure of a country affects water withdrawal. The industrial structure depends on trade openness and comparative advantages in water resources, as they reflect in capital intensity (the capital–labor ratio) and relative water abundance (*RWs*).

The *Y*_*it*_ term on the right-hand side of Eq (5) reflects the effect of production on water withdrawal (i.e., the scale effect), and the *Z*_*it*_ and Z_it_^2^ terms represent the technical effect. The terms excluding c_1_, *Y*_*it*_, *Z*_*it*_, Z_it_^2^, *Area*_*i*_ and climate variables and dummies (i.e. *Prcp*_*it*_, *Temp*_*it*_, *Tropical*_*i*_, *Dry*_*i*_, *Cold*_*i*_) on the right-hand side show the composition effects. We can divide Eq (5) into the scale effect (*Scale*_*it*_), the technique effect (*Tech*_*it*_), and the composition effect (*Comp*_*it*_):

$$Scale_{it}=\gamma_{1}Y_{it}$$ (8)

$$Tech_{it}=\gamma_{2}Z_{it}+\gamma_{3}Z_{it}^{2}$$ (9)

$$\begin{array}{c} Comp_{it}^{1}=\gamma_{4}{\left(K/L\right)}_{it}+\gamma_{5}{\left(K/L\right)}_{it}^{2}+\gamma_{6}{\left(K/L\right)}_{it}Z_{it}+\gamma_{7}T_{it}\\ +\gamma_{8}{\left(RK/L\right)}_{it}RWs_{it}T_{it}+\gamma_{9}{\left(RK/L\right)}_{it}^{2}RWs_{it}T_{it}+\gamma_{10}RZ_{it}T_{it}\\ +\gamma_{11}RZ_{it}^{2}T_{it}+\gamma_{12}{\left(RK/L\right)}_{it}RZ_{it}RWs_{it}T_{it} \end{array}$$ (10)

Eq (10) can be divided into two further parts. One is the indirect trade-induced composition effect (*OC*_*it*_), and the other is the direct trade-induced composition effect (*TC*_*it*_). These can be written as follows:

$$OC_{it}^{1}=\gamma_{4}{\left(K/L\right)}_{it}+\gamma_{5}{\left(K/L\right)}_{it}^{2}+\gamma_{6}{\left(K/L\right)}_{it}Z_{it},$$ (11)

$$\begin{array}{c} TC_{it}^{1}=\gamma_{7}T_{it}+\gamma_{8}{\left(RK/L\right)}_{it}RWs_{it}T_{it}+\gamma_{9}{\left(RK/L\right)}_{it}^{2}RWs_{it}T_{it}+\gamma_{10}RZ_{it}T_{it}.\\ +\gamma_{11}RZ_{it}^{2}T_{it}+\gamma_{12}{\left(RK/L\right)}_{it}RZ_{it}RWs_{it}T_{it} \end{array}$$ (12)

Note that when based on specification 2, Eqs (10), (11) and (12) can be rewritten as:

$$\begin{array}{c} Comp_{it}^{2}=\gamma_{4}{\left(Agri\right)}_{it}+\gamma_{5}{\left(Agri\right)}_{it}^{2}+\gamma_{6}{\left(Agri\right)}_{it}Z_{it}+\gamma_{7}T_{it}\\ +\gamma_{8}{(RK/L)}_{it}RWs_{it}T_{it}+\gamma_{9}{\left(RK/L\right)}_{it}^{2}RWs_{it}T_{it}+\gamma_{10}RZ_{it}T_{it}\\ +\gamma_{11}RZ_{it}^{2}T_{it}+\gamma_{12}{\left(RAgri\right)}_{it}RZ_{it}RWs_{it}T_{it} \end{array}$$ (13)

$$OC_{it}^{2}=\gamma_{4}{\left(Agri\right)}_{it}+\gamma_{5}{\left(Agri\right)}_{it}^{2}+\gamma_{6}{\left(Agri\right)}_{it}Z_{it}$$ (14)

$$\begin{array}{c} TC_{it}^{2}=\gamma_{7}T_{it}+\gamma_{8}{\left(RK/L\right)}_{it}RWs_{it}T_{it}+\gamma_{9}{\left(RK/L\right)}_{it}^{2}RWs_{it}T_{it}+\gamma_{10}RZ_{it}T_{it}.\\ +\gamma_{11}RZ_{it}^{2}T_{it}+\gamma_{12}{\left(RK/L\right)}_{it}RZ_{it}RWs_{it}T_{it} \end{array}$$ (15)

The effect of a 1% increase in the intensity of trade openness can be analyzed through the four paths along which the trade elasticity of water withdrawal is driven [14]. The first is the trade-induced scale effect (σ_*TS*_); the second is the trade-induced technique effect (σ_*TT*_). These effects can be derived by differentiating Eqs (8) and (9). By differentiating Eqs (8) and (9) by trade openness, we acquire:

$$\sigma_{TS}^{1}=\frac{\beta_{1}\delta_{3}T_{it}}{Z_{it}w_{it}}$$ (16)

$$\sigma_{TT}^{1}=\frac{\left(\gamma_{2}+2\gamma_{3}Z_{it}\right)\beta_{1}T_{it}}{w_{it}}$$ (17)

The other effects relate to the composition effect: the third refers to the direct trade-induced composition effect (σ_*DTS*_), and the fourth to the indirect trade-induced composition effect (σ_*ITS*_). These effects are also derived by differentiating Eqs (11) and (12):

$$\sigma_{ITC}^{1}=\frac{\gamma_{6}{\left(K/L\right)}_{it}\beta_{1}T_{it}}{w_{it}}$$ (18)

$$\sigma_{DTC}^{1}=\left(\frac{\gamma_{10}T_{it}}{\bar{Z}}+\frac{2\gamma_{11}Z_{it}T_{it}}{{\bar{Z}}^{2}}+\frac{\gamma_{12}{\left(RK/L\right)}_{it}RWs_{it}T_{it}}{\bar{Z}}\right)\beta_{it}\frac{T_{it}}{w_{it}},$$ (19)

where *RZ*_*it*_ is defined as $Z_{it}/\bar{Z}$, where $\bar{Z}$ is a world-averaged TFP value. Note that our empirical method of capturing the indirect trade-induced composition effect may also capture other effects. In particular, it may capture the portion of a country’s capital accumulation that is not trade-related. Our empirical results on the indirect trade-induced composition effect may therefore overstate the effect of trade. In calculating the direct and indirect trade-induced composition effect based on specification 2, we differentiate Eqs (14) and (15) and obtain:

$$\sigma_{ITC}^{2}=\frac{\gamma_{6}{\left(Agri\right)}_{it}\beta_{1}T_{it}}{w_{it}}$$ (20)

$$\sigma_{DTC}^{2}=\left(\frac{\gamma_{10}T_{it}}{\bar{Z}}+\frac{2\gamma_{11}Z_{it}T_{it}}{{\bar{Z}}^{2}}+\frac{\gamma_{12}{\left(RK/L\right)}_{it}RWs_{it}T_{it}}{\bar{Z}}\right)\beta_{it}\frac{T_{it}}{w_{it}}.$$ (21)

## Results and Discussion

Table 2 shows the results of the parameters in the trade equation. All parameters are highly statistically significant, and the results generally align with those of previous studies (i.e., [28], [14]).

**Table 2:** Estimates in Trade Equation.

Varibles in Eq (1)	Parameter estimates
ln(GDP_i_)	0.898***
	(163.69)
ln(GDP_j_)	0.904***
	(167.88)
ln(*Distance*_*ij*_)	-0.938***
	(-135.05)
*Language*_*ij*_	0.737***
	(34.24)
*Border*_*ij*_	0.267***
	(9.25)
*Landlocked*_*ij*_	-0.363***
	(-23.82)
Constant	-20.8***
	(-81.62)
Number of countries	196
Observations	386066
R squared	0.8644

Notes: Values in parentheses are t-values.

*** indicates significance at the 1%.

Table 3 shows the results of the parameters in the TFP equation. The coefficient of trade openness in the TFP equation is statistically significant at the 5% level. Table 4 presents the results of the parameter estimates in the growth equation. The estimated parameters of the variables of capital stock, the number of employed workers, and TFP are all statistically significant at the 1% level.

**Table 3:** Estimates in TFP Equation.

Variables in Eq (2)	Parameter estimates
*T*_*it*_	2.90**
	(1.27)
Constant	214***
	(3.22)
Observations	3528
Number of countries	117
AR(1): prob>chi^2^	0.345
AR(2): prob> chi^2^	0.173

Notes: Values in parentheses are t-values.

** and *** indicate significance at the 5% and 1%.

**Table 4:** Estimates in Growth Equation.

Variables in Eq (4)	Parameter estimates
ln *K*_*it*_	0.447***
	(11.64)
ln *L*_*it*_	0.577***
	(12.10)
ln *Z*_*it*_	0.782***
	(8.62)
Constant	0.111
	(0.22)
Observations	4181
Number of countries	130
AR (1): prob>chi^2^	0.223
AR (2): prob>chi^2^	0.139

Notes: Values in parentheses are t-values.

*** indicates significance at the 1%. Year dummies were included in the estimation, but are excluded from the table.

Table 5 shows the results of the parameter estimates in the water use equation. In Table 5, two types of specifications are examined.

**Table 5:** Estimates in Water Use Equation.

Variables	Parameter estimates			
	Dependent variable: water withdrawal		Dependent variable: water consumption	
	Specification 1:	Specification 2:	Specification 1:	Specification 2:
	Eq (5)	Eq (7)	Eq (5)	Eq (7)
*(1) Y*	0.0652***	0.0485***	0.0294***	0.0179***
	(20.41)	(10.54)	(13.90)	(8.12)
*(2) Z*	-1.65***	-0.727**	-1.16***	-0.366
	(-9.42)	(-2.30)	(-8.50)	(-1.31)
*(3) Z* ^*2*^	0.00189***	0.000800**	0.00130***	0.000398
	(8.42)	(2.27)	(7.51)	(1.32)
*(4) (K/L)*	2.67***		1.65***	
	(4.61)		(5.18)	
*(5) (K/L)* ^*2*^	-0.00181		-0.000105	
	(-0.54)		(-0.06)	
*(6) (K/L)Z*	-0.00718***		-0.00446***	
	(-6.24)		(-6.08)	
* (4) (Agri)*		9.96***		6.38***
		(3.98)		(3.48)
* (5) (Agri)* ^*2*^		-0.0998***		-0.0567***
		(-3.88)		(-3.33)
* (6) (Agri)Z*		-0.00836**		-0.00612*
		(-2.18)		(-1.99)
*(7) T*	-2.50***	-1.96***	-1.510***	-1.64***
	(-3.99)	(-4.75)	(-3.67)	(-6.21)
*(8) (RK/L)(RWs)T*	-0.324***	-0.0156	-0.191***	0.0272
	(-3.05)	(-0.06)	(-5.14)	(0.20)
*(9) (RK/L)* ^*2*^ *(RWs)T*	-0.0567*	-0.0558	-0.0451***	-0.0412
	(-1.69)	(-0.60)	(-2.69)	(-0.71)
*(10) (RZ)T*	4.16***	4.03***	2.33***	3.04***
	(4.20)	(4.21)	(3.49)	(6.17)
*(11) (RZ)* ^*2*^ *T*	-1.26***	-1.83***	-0.608**	-1.35***
	(-3.13)	(-3.79)	(-2.27)	(-5.85)
*(12) (RK/L)(RZ)(RWs)T*	0.372***	0.224**	0.240***	0.142*
	(9.15)	(2.18)	(6.68)	(1.68)
*(13) Prcp*	-0.172***	-0.207***	-0.132***	-0.183***
	(-3.38)	(-3.72)	(-3.59)	(-3.71)
*(14) Temp*	13.7***	17.4***	5.06***	6.31***
	(5.77)	(6.02)	(4.48)	(3.76)
*(15) (Temp)* ^*2*^	-0.380***	-0.506***	-0.184***	-0.214***
	(-7.37)	(-8.04)	(-6.75)	(-6.46)
*(16) Tropical*	19.0	39.9*	24.1***	32.4***
	(1.32)	(1.71)	(3.67)	(2.92)
*(17) Dry*	-42.8	-78.3***	-27.4***	-53.2***
	(-1.56)	(-3.15)	(-3.51)	(-3.05)
*(18) Cold*	86.7***	105*	-8.65	31.6
	(3.09)	(1.99)	(-0.46)	(1.01)
*(19) Area*	7.85***	10.2***	4.27***	4.83**
	(5.21)	(2.77)	(4.04)	(2.37)
Constant	230***	-26.1	228***	36.7
	(5.37)	(-0.41)	(7.61)	(0.65)
Observations	151	127	151	127
Number of countries	51	46	51	46
AR(1): prob>chi^2^	0.227	0.119	0.239	0.147
AR(2): prob>chi^2^	0.857	0.057	0.438	0.114

Notes: Values in parentheses are t-values.

*, **, and *** indicate significance at the 10%, 5%, and 1%. Year dummies are excluded from the result.

The first column in Table 5 shows that the variables and the numbers correspond to those of the coefficients in water use equations. The second and third columns contain the results of the estimation with two specifications, the dependent variable of which is water withdrawal. Similarly, the fourth and fifth columns show the estimation results, the dependent variable of which is water consumption.

First, let us examine the former results, found in the second and third columns of Table 5. In specification 1, all the estimated parameters—save for the squared term of the capital–labor ratio (*(K/L)* ^*2*^), the Tropical dummy and the Dry dummy—are statistically significant; almost all parameters other than the term of *(RK/L)* ^*2*^ *(RWs)T* exhibit statistical significance at the 1% level. In specification 2, the estimated parameters related to GDP (*Y*), agricultural output ratio (*Agri*, *(Agri)* ^*2*^), trade openness (*T*), interaction terms such as *(RZ)T* and *(RZ)* ^*2*^ *T*, the climate variables *Prcp*, *Temp*, and *(Temp)* ^*2*^, and the Dry dummy and Area are statistically significant at the 1% level. The parameters of total factor productivity (*Z* and *Z* ^*2*^) are significant at the 5% level.

In either specification 1 or 2, the estimated parameters of GDP (*Y*) are statistically significant at the 1% level. The parameters related to the capital–labor ratio (*K/L*, *(K/L)* ^*2*^, *(K/L)Z*) in specification 1 are significant except for the squared term; on the other hand, those parameters in specification 2 relate to the proportion of agricultural output (*Agri*, (*Agri)* ^*2*^, *(Agri)Z*) are statistically significant at the 1%, 1%, and 5% levels, respectively. The parameters of trade openness (*T*) are significant at the 1% level, in both specifications 1 and 2. The negative sign of the estimated parameter of trade openness indicates that it has a negative impact on the amount of water withdrawn or consumed—that is, the level of water use decreases along with an expansion in trade openness. This finding aligns with that of Hoehn and Adanu [12]. The interaction terms of relative TFP and trade openness (*(RZ)T* and *(RZ)* ^*2*^ *T*) are both statistically significant at the 1% level, in either specification 1 or 2. The climate variables of average annual temperature and its squared term are statistically significant in both specifications 1 and 2. In specification 1, the sign of the squared term of average annual temperature is negative, indicating an inverted-U relationship between annual temperature and water withdrawal. This result diverges from that of Hoehn and Adanu [12], who find the sign of the squared term of average annual temperature to be positive. Hoehn and Adanu simply interpret the positive sign of the squared temperature thus: an increase in temperature would reduce the degree of water withdrawal. On the other hand, our result implies that an increase in the average annual temperature would encourage agricultural production up to about 18.0°C in specification 1 and 17.2°C in specification 2, which would in turn increase water withdrawal; when it exceeds 18.0°C and 17.2°C, respectively, however, agricultural production would be discouraged due to the increase in the average annual temperature, which would in turn reduce water withdrawal. These results seem fairly compatible with the actual pattern of water use in agriculture.

We now examine the final sets of results in Table 5 (the fourth and fifth columns), in which water consumption is set as the dependent variable. As shown in Table 5, the overall trends vis-à-vis signs and significance share some similarities with the earlier sets of results. Although the parameters of *(K/L)* ^*2*^ and the Cold dummy in specification 1 are not statistically significant, other estimated parameters all exhibit statistical significance at the 1% level, save for the parameter of *(RZ)* ^*2*^ *T*, which is significant at the 5% level. In specification 2, the parameters of *Y*, *(Agri)*, *(Agri)* ^*2*^, and *T* are statistically significant at the 1% level. The parameters of the interaction terms—such as *(RZ)T* and *(RZ)* ^*2*^ *T—*show significance at the 1% level, whereas the parameters of *(Agri)Z* and *(RK/L)(RZ)(RWs)T* are significant at the 10% level. The coefficients of *(Temp)* ^*2*^ and *Temp* are statistically significant, and the results also suggest the existence of an inverted-U relationship between average annual temperature and the amount of water use. The inflection point is at about 13.8°C for specification 1 and 14.7°C for specification 2.

A caveat should be made here, that the results in Table 5 were obtained from a linear–linear model; when the model is transformed to log–linear, we could confirm the same result in just one specification (i.e., when the dependent variable is log of water consumption and taking log of the main variables in specification 2) of the four specifications. This suggests the possibility that the results in Table 5 are not sufficiently robust for the functional transformation, and this in turn suggests a need for further analysis.

We now turn to the effect of trade openness on water withdrawal. Table 6 shows the elasticity of the trade-induced scale effect (σ_*TS*_), the trade-induced technique effect (σ_*TT*_), the trade-induced indirect composition effects (σ_*ITS*_), and the trade-induced direct composition effects (σ_*DTS*_). These effects are calculated by substituting the parameters in Eqs (16)–(21) for the estimated values from Table 5 and the global average values of each variable. In specification 1, all parameters are statistically significant at the 1% or 5% level, irrespective of the dependent variable (water withdrawal/consumption). In specification 2, all parameters exhibit significance at the 5% level in the case of water withdrawal, while for water consumption, three parameters exhibit significance at the 5% level, save for the trade-induced technique effect (σ_*TT*_). Since σ_*TT*_ is not statistically significant, the overall trade-induced is found to be insignificant, although it does turn out to be significant if all the parameters were to be averaged.

**Table 6:** Elasticity of trade openness.

	Water withdrawal		Water consumption	
*Elasticity*	Specification 1	Specification 2	Specification 1	Specification 2
	(Eqs 16–19)	(Eqs 16, 17, 20, 21)	(Eqs 16–19)	(Eqs 16, 17, 20, 21)
*σ*_*TS*_	0.0153**	0.0153**	0.0295**	0.0295**
*σ*_*TT*_	-2.12** *	-1.10**	-3.14** *	-1.11
*σ*_*ITC*_	-1.72**	-1.23**	-2.07**	-1.74**
*σ*_*DTC*_	2.83** *	1.17**	3.67** *	1.25**
*overall*	-1.00** *	-1.52**	-1.51**	-0.491

Notes: Values in parentheses are t-values.

** and *** indicate significance at the 5% and 1%. Each elasticity is evaluated at sample means. The statistical significance of each trade-induced elasticity is calculated by taking the average of the significance of those parameters which have been used in the calculation. Similarly, the statistical significance of overall elasticity is calculated by taking the average of each trade-induced elasticity’s significance.

The results show that, first, if trade openness were to increase by 1%, water withdrawal would increase by roughly 0.015% in both specifications, while water consumption would increase by 0.030% through the trade-induced scale effect.

Second, the sign of the trade-induced technique effect is negative in both specifications 1 and 2, irrespective of the dependent variable. This indicates a negative relationship between trade openness and water withdrawal/consumption. In specification 1, the result indicates that a 1% increase in trade openness reduces the degree of water withdrawal/consumption by 2.1% (second column) and 3.1% (fourth column). Similarly, in specification 2, a 1% increase in trade openness reduces the degree of water withdrawal by 1.1% (third column). These results can be interpreted thus: an expansion in trade openness encourages people to adopt water-saving technologies (e.g., drip irrigation, water recycling, and reclamation), or it facilitates the transfer of such technologies across borders.

Third, the indirect trade-induced composition effects are all negative, whereas the direct trade-induced composition effects are all positive. These results indicate that a 1% increase in trade openness has the effect of reducing the degree of water withdrawal/consumption by changing the capital–labor ratio by 1.7% and 2.1% in specification 1 (from columns 2 and 4) and 1.2% and 1.7% in specification 2 (from columns 3 and 5). On the other hand, a 1% increase in trade openness has the effect of increasing water withdrawal by 2.8% and 3.7% in specification 1 (from columns 2 and 4) and by 1.2% and 1.3% in specification 2 (from columns 3 and 5). These changes can be interpreted as outcomes stemming from the comparative advantage effect. It should be noted that our analysis is confined to the short term; this means that there remains the possibility that the indirect trade-induced composition effect may capture an effect other than that induced by trade (i.e., capital accumulation). This would especially be the case for developing countries, and it might provide an explanation for the results in column 5 of Table 6 (specification 2 of the water consumption model), wherein the magnitude of the indirect trade-induced composition effect is fairly large.

Finally, the overall effect (= σ_*TS*_ + σ_*TT*_ + σ_*ITS*_ + σ_*DTS*_) induced by trade can be calculated as –1.0 for the water withdrawal model in specification 1, –1.52 for the same model in specification 2, and –1.51 for the water consumption model in specification 1. This means that the overall effect of a 1% increase in trade openness reduces the degree of water withdrawal/consumption by 1.0–1.5%, on average. This result indicates that international bilateral trade could promote efficient water use, through the diffusion of water-saving technologies and the principle of comparative advantage.

## Conclusion

This study examined the effect of trade openness on water withdrawal and consumption. Our results indicate that increased trade openness may reduce water consumption via the scale, technique, and composition effects induced by trade. While trade positively affects water withdrawal/consumption through the trade-induced scale effect, the trade-induced technique and indirect composition effect—both of which exhibit a negative sign—counteract the scale effect and direct composition effect, resulting in reduced water consumption. The results show that trade openness has a favorable effect on water use efficiency; however, it is fair to say that this favorable effect would be lost if a country were to erect a barrier to technological development accompanied by trade liberalization.

In the current study, despite limited data availability, we evaluated the effect of trade openness on water use as precisely as possible by dealing with an inherent endogeneity problem; this is its net contribution. As bilateral international trade is an important factor not only in terms of economic development but also water use, we must further analyze the relationship between trade and water use and accumulate relevant data. Further work is needed, for example, to undertake theoretical and empirical examinations of the determinants of water use and panel data analyses of the impact of water scarcity on economic growth, while focusing on its effect on trade.
